# Microbial Diversity and Bioactive Compounds in Dried *Lycium barbarum* Fruits (Goji): A Comparative Study

**DOI:** 10.3390/molecules28104058

**Published:** 2023-05-12

**Authors:** Katarzyna Rajkowska, Anna Otlewska, Natalia Broncel, Alina Kunicka-Styczyńska

**Affiliations:** 1 Institute of Fermentation Technology and Microbiology, Faculty of Biotechnology and Food Sciences, Lodz University of Technology, Wólczańska 171/173, 90-530 Łódź, Poland; anna.otlewska@p.lodz.pl (A.O.); nataliagniada10@gmail.com (N.B.); 2 Bionanopark Ltd., Dubois 114/116, 93-465 Łódź, Poland; 3 Department of Sugar Industry and Food Safety Management, Faculty of Biotechnology and Food Sciences, Lodz University of Technology, Wólczańska 171/173, 90-530 Łódź, Poland; alina.kunicka@p.lodz.pl

**Keywords:** goji, bioactivity, high-throughput sequencing, geographic region, natural dried goji fruit, freeze-dried goji fruit, microbial quality, antioxidant activity

## Abstract

This study compares the microbial diversity and content of bioactive compounds in dried goji berries available on the Polish market to those of the most highly valued goji berries from the Ningxia region in China. The content of phenols, flavonoids, and carotenoids were determined, as well as the antioxidant capacities of the fruits. The quantitative and qualitative composition of the microbiota inhabiting the fruits was assessed using metagenomics by high-throughput sequencing on the Illumina platform. The highest quality was demonstrated by naturally dried fruits from the Ningxia region. These berries were characterized by a high content of polyphenols and high antioxidant activity, as well as high microbial quality. The lowest antioxidant capacity was shown by goji berries cultivated in Poland. However, they contained a high amount of carotenoids. The highest microbial contamination was found in the goji berries available in Poland (>10^6^ CFU/g), which is important in terms of consumer safety. Despite the widely accepted benefits of consuming goji berries, both the country of cultivation and the preservation method may influence their composition, bioactivity, and microbial quality.

## 1. Introduction

The popularity of functional foods and medicinal plants that can prevent or inhibit the development of diseases has increased significantly in recent years. This increase is associated with the rising interest of consumers and food producers in plants with high contents of bioactive ingredients—often referred to as superfoods for their beneficial effects on health and well-being.

*Lycium* plants belong to the *Solanaceae* family and occur naturally in the arid and semi-arid regions of Eurasia, Africa, and North and South America [1]. Worldwide, 31 species of *Lycium* have been reported as medicines or foods, but to date two species are best known for their chemical composition and health effects, namely, *Lycium barbarum* L. and *Lycium chinense* Mill. *Lycium* fruits, also known as wolfberries, goji berries, or barbary wolfberries, have been used in traditional Chinese medicine for more than 2000 years [2]. Goji berry extracts and infusions have been traditionally used as ingredients in nonalcoholic and alcoholic beverages that have beneficial effects on the eyes, kidneys, and liver, expressing anti-aging properties. Wolfberries are considered superfruits for their high content of nutrients with high biological activity, including polysaccharides, carotenoids, and phenolics. The fruits are also an excellent source of macronutrients, including vitamins such as riboflavin, thiamine, ascorbic acid, mineral elements (potassium, sodium, phosphorous, magnesium, calcium, and selenium), and 16 various amino acids [2,3].

Valuable and well-studied polysaccharides occur in *L. barbarum* fruits in the form of branched, water-soluble complexes, constituting 5–8% of the dried fruit and 1–2.5% of the fresh berry material. Six types of monosaccharides form part of the complexes: arabinose, rhamnose, xylose, mannose, galactose, glucose, and galacturonic acid [3]. Due to the fact of their antioxidant properties, *L. barbarum* polysaccharides reduce oxidative stress related to age, exhaustive exercise, and alcohol consumption [4,5,6]. Additionally, it has been reported that polysaccharide complexes have neuroprotective, radioprotective, antitumor, anti-osteoporosis, hypolipidemic, and antihyperglycemic properties [2,5,7].

The protective and antioxidant activity of goji berries also results from the presence of phenolic compounds. Different types of polyphenols have been isolated from goji berries, including phenylpropanoids, coumarins, lignans, flavonoids, isoflavonoids, and chlorogenic acid derivatives [8]. Phenolic compounds can relieve diseases caused by oxidative stress, such as cardiotoxicity and acute lung injury. Moreover, phenolics have antidiabetic, anti-inflammatory, neuroprotective, and anticancer potential [9]. *Lycium* phenolic compounds can also support intestinal health by improving the richness and diversity of gut microbiota, promoting the growth of probiotic *Bifidobacterium* and *Lactobacillus* species, and increasing the production of short-chain fatty acids [8,9].

Another important group of active compounds in goji berries are carotenoids, which are responsible for the characteristic orange-reddish color of the fruits. The predominant carotenoid is zeaxanthin, which is found mainly in the form of dipalmitin. It accounts for one-third to one-half of all carotenoids. The total carotenoid content in dried fruits is 0.03–0.5%. Zeaxanthin accumulates in the macula, which it protects from degradation by UV light, and displays neuroprotective properties on the retina of diabetic animals [10]. The other carotenoids present in goji berries but in lower concentrations are lutein, β-carotene, violaxanthin, and cryptoxanthin [11].

Goji plants are widely grown in East Asia, specifically in the western part of China, Tibet, Mongolia, Japan, Korea, and Taiwan. Recently, goji berries have also gained popularity on the European market [10]. *Lycium* spp. has been cultivated for centuries in the Ningxia region of China and has the status of “daodi”. Daodi products come from specific geographical regions with special natural conditions. They are widely recognized for their highly stable quality and reliable effectiveness [12]. Due to the fact of their ease of cultivation and growing market demand, new goji plantations are being developed in many European countries, including Poland. Comparing the quality and health-promoting value of goji berries from the Ningxia region and Polish cultivation is, therefore, important from both the cognitive and consumer point of view.

Previously, we investigated the microbial quality and bioactivity of goji fruit extracts obtained using various solvents and green extraction methods [13]. Since the results indicated the possibility of a high degree of contamination of dried goji berries with microorganisms, we decided to extend our research towards identifying the dominant microbiota inhabiting these fruits.

Goji berries, like most fresh fruits, are subject to rapid deterioration due to the fact of their high water content (more than 80%), which offers a reservoir for microorganisms, including pathogens. Studies have shown that fresh fruits may be inhabited by *Salmonella enterica*, *Escherichia coli* O157:H7, *Bacillus cereus*, *Listeria monocytogenes*, and *Pseudomonas* spp. [14]. Extending the shelf life of fruits by drying them reduces water activity and inhibits the growth of microorganisms. However, such preserved fruits are exposed to contamination when dried in unhygienic conditions [15]. Dried fruits can be carriers of both nonpathogenic and pathogenic microflora, namely, bacteria, including *Streptococcus* spp., *Pseudomonas* spp., *Enterobacter* spp., *Bacillus* spp., *Salmonella* spp., *Cronobacter* spp., *Staphylococcus aureus*, *Escherichia coli*, and *Proteus mirabilis*, and fungi, including *Aspergillus* spp., *Penicillium* spp., and *Fusarium* spp. [16,17]. To protect consumer safety, it is important to undertake research not only on the levels of contamination in dried goji berries but also on the structure of the microbial community.

*Lycium* sp. fruits are collected in summer and autumn. They are traditionally dried in the shade and then in the sun, so that the pulp is soft and the outer skin is hard [2]. Currently, goji fruits are available in freeze-dried, frozen, powdered, and tablet form. The recommended daily intake of dried fruit is 6–18 g [18]. This has implications for consumer safety in light of the level of microbial contamination and microbiota composition of goji berries.

The aim of this study was to determine the diversity of microbiota and the content of bioactive compounds (polyphenols, flavonoids, and carotenoids) in dried goji berries available in Poland compared to the most highly valued berries from the Ningxia region. To the best of our knowledge, this is the first study considering the composition of goji berry microbiota.

## 2. Results and Discussion

**Figure 1:**
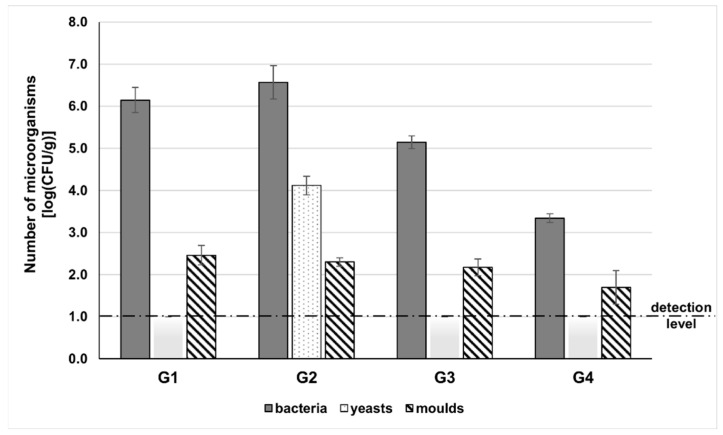
The level of microorganisms contaminating goji berries of various origins: G1—natural dried goji berries from China, packed in Poland; G2—freeze-dried goji berries from Poland; G3—natural dried goji berries from Ningxia, China; G4—freeze-dried goji berries from Ningxia, China.

This study compared the level of microorganisms contaminating goji berries of various origins: G1—natural dried goji berries from China, packed in Poland; G2—freeze-dried goji berries from Poland; G3—natural dried goji berries from Ningxia, China; and G4—freeze-dried goji berries from Ningxia, China. The level of microbial contamination varied (Figure 1). In general, bacteria were the predominant group of microorganisms. The highest numbers of bacteria were found in freeze-dried fruits grown in Poland (G2) and natural dried berries from China, packed in Poland (G1) (3.7 × 10^6^ and 1.4 × 10^6^ CFU/g, respectively). Only in berries cultivated in Poland (G2) were significant amounts of yeasts found, at 10^4^ CFU/g, whereas in all other fruits the amounts of yeasts were below the detection level of 10 CFU/g. The lowest number of microorganisms was estimated in freeze-dried goji berries from Ningxia (G4) in which the number of bacteria and molds were 2.2 × 10^3^ and 5.0 × 10^1^ CFU/g, respectively. In the other fruits tested, the amounts of mold were higher, ranging from 1.5 × 10^2^ to 2.9 × 10^2^ CFU/g (Figure 1). The high bacterial level, reaching even 6.5 log units per 1 g of berries (G2), may raise the question of the composition of the microbial community. Metagenomics revealed the presence of microbial species residing on the berries regardless of the stage of plant growth, area of cultivation, and method of fruit processing.

There is little information on the microbial quality of goji berries, either at the stages of fruit processing and preservation or in terms of the finished product. Most research discusses the quality of goji berries in terms of preserving their shelf life [19,20]. There are few studies concerned with the microbial quality in relation to the presence of food-borne pathogens, which can not only cause spoilage and shorten the shelf life but also be dangerous to the consumer. The *Codex Alimentarius* [21] does not define microbiological limits for dried fruits; however, it states that they should not contain any pathogens or any toxic substances of microbiological origin.

The total microbial abundance was significantly different for each sample of dried goji fruits. The highest number of bacterial OTUs was found for natural dried fruits from China, packed in Poland (G1), and natural dried fruits from Ningxia (G3) amounting to 14,060 and 2896, respectively. These values were significantly lower in freeze-dried fruits (G2 and G4), in the range of 17–101 OTUs. In contrast, the fungal total abundance was very high (4816–85,424 OTUs) regardless of the fruit’s method of preservation, with the highest (85,424 OTUs) for the sample of freeze-dried berries (G4) originating from China and packed in Poland.

**Figure 2:**
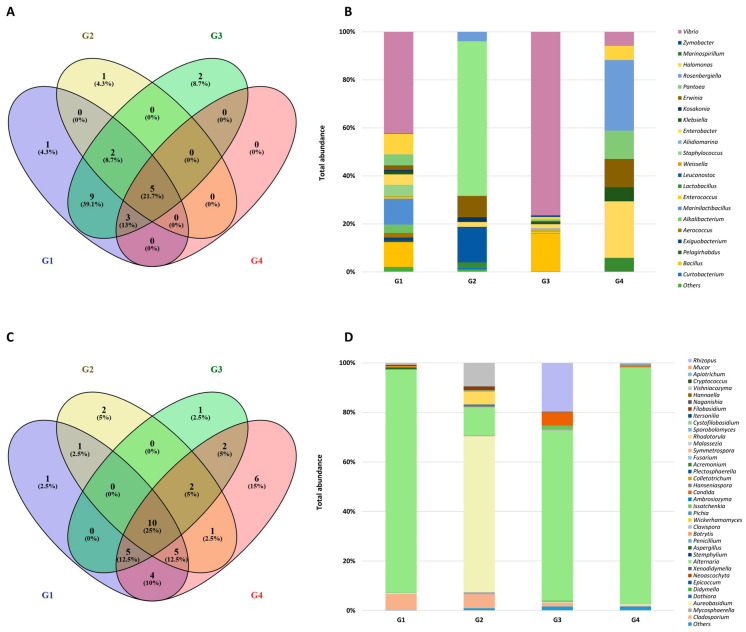
Venn diagram showing the overlap of bacterial genera and the relative abundance of major bacterial genera (percentage of the total OTUs) (**A**,**B**) and fungal genera (**C**,**D**) in goji berries of various origins: G1—natural dried goji berries from China, packed in Poland; G2—freeze-dried goji berries from Poland; G3—natural dried goji berries from Ningxia, China; G4—freeze-dried goji berries from Ningxia, China.

The high-throughput sequencing data showed the prevalence of 23 bacterial genera, belonging to three different classes. The air-dried goji berries from China (G1) were characterized by the highest biodiversity (H 2.06), while the Shannon diversity index for the remaining samples (G2–G4) was significantly lower, in the range of 0.61–1.23. Regardless of the origin and preservation methods, bacteria belonging to the phylum *Proteobacteria* were dominant and constituted from 63.7% to 94.1% of the total OTUs (Figure 2). Among this phylum, the genera *Pantoea* (0.7–64.4%), *Rosenbergiella* (0.03–29.4%), *Enterobacter* (1.7–23.5%), and *Erwinia* (0.4–11.8%) represented the *Enterobacteriaceae* family and were identified in all samples (Figure 2B). In the freeze-dried fruits (G2 and G4), *Enterobacteriaceae* accounted for over 80% of *Proteobacteria* sequences.

The presence of *Pantoea* sp. and *Rosenbergiella* sp. was reported on the surface of raw goji berries from China by Huang et al. [22]. These bacteria were the prevailing genera in the majority of the tested samples. Moreover, when the microbiome was dominated by *Pantoea*, the goji berries showed a higher rate of decay. *Pantoea* was also found in dried and packaged goji fruits [23], which may indicate that these bacteria are part of the indigenous microbiome. The species detected on the freeze-dried goji berries, *Pantoea agglomerans*, is associated with both detrimental and beneficial activities [24]. *P. agglomerans* is both an epiphytic and endophytic bacteria. It occurs in the soil, air, and above all in dusts (plant, wood, and other agricultural environments), causing infections in people who are particularly exposed to the inhalation of organic dusts. Simultaneously, *P. agglomerans* produces bioactive compounds, which inhibit the development of phytopathogens and promote plant growth [25]. It has been reported that low-moisture foods may be contaminated with pathogenic bacteria belonging to the *Enterobacteriaceae* family, including *Salmonella* spp., Shiga-toxigenic *Escherichia coli*, and *Cronobacter sakazakii* [26]. Pathogens can remain active in these types of food for at least months, posing a serious threat to consumer safety [27].

In the natural dried fruits (G1 and G3), *Vibrio* spp. bacteria were detected, accounting for 42% and 76% of the bacteria pool, respectively (Figure 2B). The most dominant species was *Vibrio metschnikovii*, while *V. alginolyticus*, *V. harveyi*, *V. natriegens*, and *V. fluvialis* were also identified. *Vibrio* sp., mainly *Vibrio metschnikovii*, colonize a variety of aquatic environments, such as streams, lakes, marine water, and wastewater. *Vibrio* spp. have been isolated from cattle, swine, horses, and poultry, which indicates that they may be zoonotic microorganisms and can be transmitted to humans via the food chain [28]. However, it should be noted that infections by *Vibrio metschnikovii* are rather rare, in contrast to those caused by *V. vulnificus*, *V. parahaemolyticus*, and *V. cholerae* [29].

*Firmicutes* accounted for 5.9–34.2% of the total bacteria, with the highest percentage found in the natural dried goji berries from China (G1 and G3). In both these samples, there were high proportions of sequences annotated to spore-forming bacteria belonging to *Bacillus* (10.3 and 15.7%), followed by *Marinilactibacillus*, *Staphylococcus*, *Alkalibacterium*, *Aerococcus*, and *Exiguobacterium*. Among *Firmicutes*, only *Lactobacillus* was present in all samples, regardless of the preservation method and country of origin (Figure 2A,B). *Lactobacillus* was also the only bacterial genus that belonged to this phylum detected in the sample of freeze-dried goji berries from Ningxia (G4) and one of three bacterial genera that were identified in fruits cultivated in Poland (G2) (along with *Leuconostoc* and *Curtobacterium*).

In the tested goji berries, 38 fungal genera belonging to 15 classes were identified, with the highest biodiversity expressed in the Shannon index (H) found in goji grown in Poland (G2) (H 1.35) and in fruits from Ningxia G3 (H 1.03). Molds of the genus *Alternaria* were detected in all samples, irrespective of the country of cultivation and the drying method. However, the highest percentages were noted in the fruits from China (90.2% for G1 and 95.4% for G4). In the goji berries cultivated in Poland (G2), there was a much lower percentage of *Alternaria* sp. (11.5%) than in the other samples (Figure 2D). In G2, *Aureobasidium* spp. dominated, constituting almost 63% of the total OTUs, followed by *Cladosporium* sp. (6.4%) and then the yeasts *Vishniacozyma* sp. (9.5%) and *Rhodotorula* sp. (5.0%). Both *Aureobasidium* spp. and *Cladosporium* spp. were detected in the remaining samples (G1, G3, and G4), but their amounts were much lower, in the ranges 0.4–0.6% and 0.3–5.6%, respectively. *Rhizopus* and *Aspergillus* were identified only in the three samples from China (Figure 2C).

There are few reports on pathogenic fungi in raw and dried goji berries. However, the contamination of goji fruits begins during harvesting and even earlier. It continues as a result of inappropriate drying and storage conditions, especially high temperatures and a high relative humidity [30]. The main pathogenic fungi causing postharvest rot of fresh berries include *Alternaria alternata*, *Alternaria gaisen*, *Cladosporium* spp., *Fusarium oxysporum*, and *Penicillium oxalicum* [31,32]. In air-dried goji fruits with mildew rot symptoms, *Alternaria alternata* and *Torulaspora delbrueckii* have been detected [33]. As reported previously [34], goji fruits may also be contaminated by fungi, including heat-resistant fungi. The presence of fungi and the possible accumulation of mycotoxins could be a significant problem, not only due to the fact of microbial spoilage during storage but also because of the potential health risk to the consumer.

Goji berries are prepared and consumed in various ways. They may be eaten raw, used in soups and smoothies, added to yogurts and cookies, or mixed with tea. Juice, wine, and tinctures are also prepared from goji berries [2,18]. Traditionally, dried goji berries are cooked before they are consumed. They are commonly used in China as a condiment for food or as tea infused with boiling water [18]. Due to the high levels of microbial contamination and possible occurrence of pathogenic strains in the examined goji berries, it seems advisable to use heat treatment before consumption. In addition, boiling may contribute to an increase in the amount of bioactive compounds by softening the plant tissues and facilitating extraction from the cell matrix [35]. On the other hand, heat treatment may degrade these compounds. The effects depend on the cooking technique used. Goji berries in a low-processed state possess prebiotic potential and may be used in foods such as yogurt. The prebiotic effect of *Lycium* fruits is probably related to the utilization of their polysaccharides and polyphenols by some probiotic bacteria, and to the inhibition of the growth of antagonistic bacteria in the gut [36,37]. A pronounced antibacterial effect was demonstrated by dried goji berries and their water extract in a milkshake, which may offer a method of extending the shelf life of this type of product [38].

In our study, the bioactivity of various goji berries was also determined, i.e., their antioxidant activity and the content of polyphenols and flavonoids in both water and ethanol extracts. The alcoholic extracts showed significantly higher antioxidant activity than the water extracts, at 43.5–46.5 μmols and. 39.7–41.6 μmols Trolox equivalents/g (Table 1). However, the ethanol extracts contained less polyphenols and slightly more flavonoids than the water extracts. The total polyphenolic content was 24.5–35.9% lower in the alcoholic extracts, and these differences were statistically significant. The highest concentration of polyphenols was found in the natural dried goji fruits from Ningxia (G3). More polyphenols were found in the natural dried berries (G1 and G3) than in the freeze-dried berries (G2 and G4). Similarly, the natural dried fruits contained more flavonoids. The highest concentration of flavonoids was determined in natural dried berries from China, packed in Poland (G1). Despite the differences in the content of polyphenols and flavonoids, the ethanol extracts of the natural dried goji berries originating from China and packed in Poland (G1) and of the natural dried and freeze-dried fruits from Ningxia (G3 and G4) showed comparable antioxidant activity (Table 1). The water and ethanol extracts from the freeze-dried goji berries cultivated in Poland (G2) showed a 4.6% and 6.5% lower antioxidant capacity, respectively.

**Figure 3:**
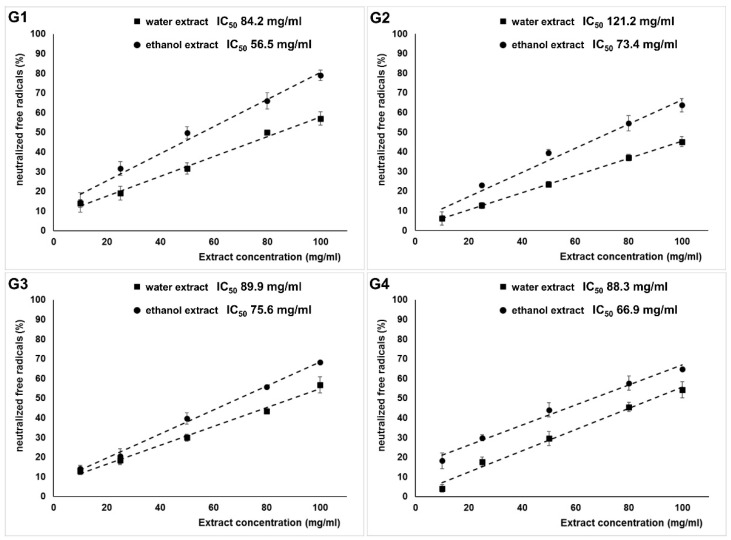
The radical-scavenging activity of goji berries: G1—natural dried goji berries from China, packed in Poland; G2—freeze-dried goji berries from Poland; G3—natural dried goji berries from Ningxia, China; G4—freeze-dried goji berries from Ningxia, China.

Consequently, freeze-dried fruits grown in Poland (G2) were characterized by the lowest ability to neutralize ABTS^•+^ radicals. The IC_50_ values were 121.2 mg/mL for the water extract and 73.4 mg/mL for the ethanol extract (Figure 3). The highest neutralizing activity was found in natural dried goji berries from China, packed in Poland (G1). As with antioxidant capacities, a higher ABTS radical-scavenging activity was found in ethanol extracts than in water extracts.

According to the data in the literature, the total content of phenols and flavonoids, as well as the antioxidant activity of *Lycium* berries, vary significantly, depending on the country of harvest, environmental conditions, cultivars, and the extraction method used. One of the lowest values for phenols was found for fruits collected in Romania (3.31–5.76 mg GAE/g), while berries cultivated in Italy presented a higher phenolic content (7.69–23.49 mg GAE/g) [39]. Compared to some samples from Italy, berries from Serbia [40] and Uzbekistan [41] were characterized by rather low polyphenol content, at 1.62 mg and 10.57–12.47 mg GAE/g, respectively. The amount of polyphenols in fruits from China was within a similar range, at 7.61–12.12 mg GAE/g [41], while as much as 109.72 mg GAE/g of polyphenols were extracted from goji berries cultivated in Greece, depending on the solvent [42]. The results obtained in this study for total phenolic content are comparable with those obtained for goji berries collected in Romania [39], and one and a half to three times higher than reported for fruits from the Ningxia region (2.17–4.48 mg GAE/g) [43]. Interestingly, in contrast to the published data, in our study the concentration of polyphenols was lower in the ethanol extracts than in the water extracts, which may be due to the use of 50% ethanol and the high solubility of polyphenols in water. The use of 50% ethanol and water as extracting solvents could have resulted in the extraction of different compounds or different extraction efficiencies of various groups of compounds contributing to the antioxidant activity.

The results obtained in this study show a relatively low flavonoid content (0.5–0.9 mg CAE/g), regardless of the region of goji cultivation, preservation method, and extraction conditions. Higher contents of flavonoids were found in goji berries from Uzbekistan (1.18–2.70 mg CAE/g), Serbia (2.14 mg of hyperoside equivalent/g), and China (1.19–2.60 mg CAE/g) [40,41]. Other researchers showed that fruits from the Ningxia region in China contained more flavonoids—up to 3.16 mg CAE/g [43], which is three and a half to six times more than in our study. Consequently, these fruits showed higher antioxidant activity than in our study, i.e., 53.92–64.38 µmol TE/g vs. 39.8–41.3 μmol TE/g.

In our study, to determine the carotenoid content in the goji berries, ethyl lactate was used. The goji berries from Ningxia (G3 and G4) were characterized by identical levels of carotenoids, irrespective of the form of preservation (Table 1). The difference in the carotenoid contents in the fruits cultivated in Poland (G2) and those originating from the Ningxia region was not statistically significant. The lowest carotenoid content was found in the natural dried goji berries from an unspecified region in China, packed in Poland (G1), at 7.3 mg/g.

Fruits from the Ningxia region in China have been reported to contain surprisingly low levels of carotenoids, in the range of 0.21–0.23 mg/g. Fruits from Uzbekistan contained 0.81 mg/g [41], and fruits from Serbia contained 0.42 mg/g [40]. Other berries from China were characterized by a higher content of carotenoids (1.19–2.60 mg/g), although this was still up to eight times lower than in our study. These differences were probably due to the use of ethyl lactate, which contributed to a significant increase in the extraction yield.

**Figure 4:**
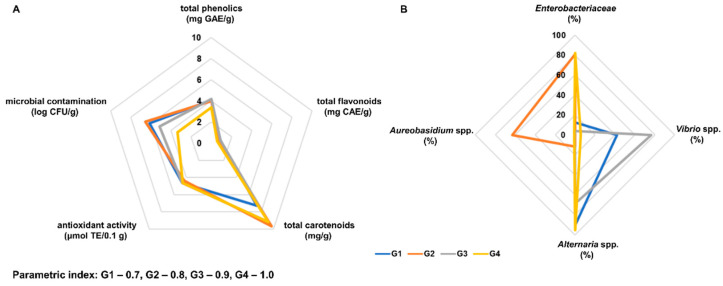
Radar chart comparing microbial contamination and bioactivity profile (**A**) and predominant microorganisms (**B**) in goji berries available in Poland and from the Ningxia region: G1—natural dried goji berries from China, packed in Poland; G2—freeze-dried goji berries from Poland; G3—natural dried goji berries from Ningxia, China; G4—freeze-dried goji berries from Ningxia, China.

Overall, the dried goji fruits available on the Polish market were heterogeneous. The berries originating from China and packed in Poland (G1) were characterized by statistically lower content of carotenoids compared to fruits cultivated in Poland and the most valued goji from the Ningxia region (Figure 4A). Although no statistically significant differences were found between the examined goji in the content of polyphenols and flavonoids, the fruits cultivated in Poland (G2) were characterized by statistically lower antioxidant activity. Distinct differences were observed in the level of microbial contamination and the structure of the dominant microbiota. However, the structure of the microbiota was more strongly dependent on the preservation method than the region of goji cultivation. The dominant group of bacteria in the natural dried fruits from China (G1 and G3) was *Vibrio* spp., and in the freeze-dried berries grown in Ningxia (G4) and in Poland (G2) the *Enterobacteriaceae* family dominated. *Alternaria* spp. molds were dominant in all goji fruits originating from China (G1, G3, and G4), while in berries cultivated in Poland (G2) *Aureobasidium* spp. prevailed (Figure 4B). The high amount of microorganisms in goji available in Poland (G1 and G2) is worrying and may raise concerns in terms of consumer safety. Goji berries from the Ningxia region showed the highest microbial quality and biological activity (Figure 4A). The parametric indexes determined for fruits originated from Ningxia were 0.9 for natural dried berries (G3) and 1.0 for freeze-dried berries (G4), reaching the maximum value for this index. The lowest index value was calculated for natural dried goji berries from China packed in Poland (G1), at 0.7 (Figure 4).

The results presented in this study may be useful in targeting goji with different characteristics for specific purposes. According to Yao et al. [12], based on metabolomic profiling of 51 goji samples from the four main production areas of different climatic regions in China, no specific production area is superior to other areas. Instead, the authors found that species affiliation was crucial for explaining the differences. *L. chinense* fruits were characterized by much lower sugar content and higher antioxidant activity than *L. barbarum*. These results indicate the possibility of using goji from different regions for different purposes, e.g., large fruits can be marketed as fresh fruits, goji with high sugar content can be used for conserved food, and fruits with high antioxidative activity can be used for medicinal purposes. In contrast, Mocan et al. [39] found differences in the phytochemical profiles of goji berries that depended not only on the genotypes, but also on pre-harvest practices, cultivar, environmental conditions, and the stage of maturity at harvest. In the light of these findings and the results presented in this study, it can be assumed that the differences in the bioactivity profiles of the tested goji berries resulted primarily from the country of cultivation and the related different climatic conditions but also probably from the different cultivars.

## 3. Materials and Methods

### 3.1. Goji Berries

Four different *Lycium barbarum* L. berries were used in the study: natural dried goji berries from an unspecified region in China, packaged and distributed in Poland by Radziowi Sp. z o.o. (Częstochowa, Poland) (G1); freeze-dried goji berries cultivated, processed, and packaged in Poland by Coactum Sp. z o.o. (Cracow, Poland) (G2); natural dried goji berries from the Ningxia region in China (G3); freeze-dried goji berries from the Ningxia region in China (G4). Sun-dried and freeze-dried goji berries from the Ningxia region were obtained courtesy of NingXia Senmiao Technology and Development Co., Ltd. (Chengdu, China).

### 3.2. Determination of the Total Number of Microorganisms in Goji Berries

Ten grams of goji berries were weighed and transferred to 90 mL of 0.1% peptone water solution. The samples were homogenized for 2 min, and ten-fold dilutions were prepared. Enumeration of microorganisms was performed using the pour plate method with plate count agar (enzymatic digest of casein: 0.5%, yeast extract: 0.25%, dextrose: 0.1%, agar: 1.5%; pH: 7.0; Merck KGaA, Darmstadt, Germany) for bacteria and dichloran glycerol agar (dextrose: 1.0%, dichloran: 0.0002%, magnesium sulfate: 0.05%, monopotassium phosphate: 0.1%, pepton: 0.5%, chloramphenicol: 0.01%, agar: 1.5%, glycerol: 18%; pH: 5.6; Merck KGaA, Darmstadt, Germany) for fungi. The plates were incubated for 24–48 h (bacteria, yeast) and up to 5 days (molds) at 30 °C. The results are expressed as colony-forming units per 1 g of goji fruits (CFU/g) as the arithmetic mean from three independent experiments.

### 3.3. Identification of Goji Berries Microbiota

#### 3.3.1. DNA Extraction

Total genomic DNA was extracted directly from goji berries (G1–G4) using a DNeasy PowerFood Microbial Kit (Qiagen, Venlo, the Netherlands), according to the manufacturer’s protocol with some modifications. The goji berries were weighed (250 mg of each sample) and ground with a lysing matrix (1.4 mm ceramic spheres, 0.1 mm silica spheres, 4 mm glass bead). The quantity and quality of the isolated DNA were measured using a Qubit 2.0 Fluorometer (Invitrogen/Life Technologies, Carlsbad, CA, USA).

#### 3.3.2. PCR Amplification and High-Throughput Sequencing

Amplification of the V3–V4 region of the 16S rRNA was carried out with the universal primer set 341F and 785R specific for bacteria [44]. The fungal ITS region was amplified with ITS1F12 (forward) and ITS2 (reverse) primers [45]. PCR reactions for both the 16S rRNA gene and the ITS region were performed under the same conditions with a Q5 Hot Start High Fidelity 2x Master Mix (New England BioLabs Inc., Ipswich, MA, USA): initial denaturation at 95 °C for 2 min, followed by 25 cycles of denaturation at 95 °C for 30 s, annealing at 55 °C for 30 s, and extension at 72 °C for 1 min, with a final extension at 72 °C for 5 min. The PCR products were purified using AMPure XP magnetic beads (Beckman Coulter Life Sciences Inc., Indianapolis, IN, USA). The amplicon libraries were indexed using a Nextera Index Kit (ThermoFisher Scientific, MA, Waltham, USA) following the manufacturer’s protocol. High-throughput sequencing was performed by Genomed S.A. (Warsaw, Poland) on a MiSeq sequencer (Illumina, Inc., San Diego, CA, USA) in paired-end technology (PE, 2 × 250 nt).

#### 3.3.3. High-Throughput Sequencing Data Analysis

A preliminary data analysis was performed using a MiSeq System (Illumina, Inc., San Diego, CA, USA) with MiSeq Reporter (MSR) v. 2.6 software (Illumina, Inc., San Diego, CA, USA). Classification of the readings was processed by the QIIME 2 bioinformatic platform (https://qiime2.org/ (accessed on 19 December 2022)) based on the SILVA v. 138 database for bacteria and UNITE v. 8.2 database for fungi [46]. The analysis comprised the following steps: removal of adapter sequences (cutadapt program), evaluation of the quality of the readings, removal of low-quality sequences (cutadapt program), connecting paired sequences (SeqPrep algorithm), clustering based on the selected database of the reference sequences (uclust algorithm), and removal of the sequence chimeras (usearch61 algorithm) [47,48]. Operational taxonomic units (OTUs) were assigned to taxa in the selected database of reference sequences. The diversity of the microbial community in the goji berries was analyzed using the Shannon index, an alpha diversity estimator calculated by Mothur v. 1.30.1. The difference in microbial genera between samples were presented on Venn diagrams (Venny 2.1, https://bioinfogp.cnb.csic.es/tools/venny/ (accessed on 23 January 2023)).

### 3.4. Goji Berries Extracts

The fruits were ground to a powder, and 1 g of each sample was suspended in 10 mL of water (water extract) or in 10 mL of 50% ethanol (ethanol extract). Extraction was carried out for 30 min in an ultrasonication bath (Sonorex, Bandelin Electronic, Berlin, Germany) at 30 °C. After cooling, the samples were centrifuged at 4500 rpm for 15 min, and the supernatants were collected. The extracts were stored at 4 °C in the dark. The total phenolic content, total flavonoid content, and antioxidant activity of the extracts were determined.

### 3.5. Antioxidant Activity

The ABTS radical-scavenging activity of the goji berry extracts was evaluated according to the method described by Re et al. [49]. ABTS radicals (ABTS^•+^) were obtained in a reaction of 7 mM ABTS (2,2′-azino-bis(3-ethylbenzothiazoline-6-sulfonic acid) diammonium salt, Roche Diagnostics, Mannheim, Germany) solution with 2.45 mM potassium peroxodisulfate (Sigma-Aldrich, St. Louis, MO, USA) in the dark at room temperature for 16 h. Then, to 1.0 mL of ABTS^•+^ solution, previously diluted to an absorbance of 0.7 at 734 nm, 10 µL of goji extracts or Trolox (6-hydroxy-2,5,7,8-tetramethylchromane-2-carboxylic acid, Sigma-Aldrich, St. Louis, MO, USA) standards was added. The mixture’s absorbance was measured after 1 min at 734 nm against appropriate solvent blanks. The antioxidant activity was calculated based on the calibration curve for Trolox. The results were expressed as μmol of Trolox equivalents per 1 g of goji fruits as the arithmetic mean from three independent experiments.

The percentage of the ABTS^•+^ free radicals neutralization was determined for different concentrations of extracts ranging from 10 to 100 mg/mL. Mixtures of 1 mL of ABTS^•+^ solution and 10 µL of the extracts were incubated for 15 min at room temperature in the dark, and the absorbance was measured at 734 nm. The percentage of ABTS free radical neutralization was calculated according to the following equation:

%ABTS^•+^ scavenging activity = ((A_control_ − A_sample_)/A_control_) × 100, (1)

where A_control_ is the absorbance of the ABTS^•+^ solution at 734 nm, and A_sample_ is the absorbance of the test sample at 734 nm. The activity of the neutralizing ABTS^•+^ radicals was expressed as the IC_50_ value, i.e., the concentration of the extract at which 50% of ABTS^•+^ free radicals were neutralized.

### 3.6. Total Phenolic Content

The total phenolic content was determined in a 96-well microplate using Folin–Ciocalteu (Sigma-Aldrich, St. Louis, MO, USA) reagent, according to the method described by Zhang et al. [50]. To 20 μL of goji berries extracts or gallic acid standards was added 100 μL of Folin–Ciocalteu reagent. The solution was mixed well. After 5 min, 80 µL of 7.5% sodium carbonate solution was added. The solution was mixed and left in the dark at room temperature for 2 h. The absorbance was measured at 750 nm with a spectrophotometric microplate reader (Asys UVM 340, Biochrom, Cambridge, UK) against the appropriate solvents (water and 50% ethanol). The total phenolic content in the analyzed extracts was calculated based on the equation of the gallic acid calibration curve. The results are expressed as the arithmetic mean from 3 independent experiments in milligrams of gallic acid equivalents per 1 g of goji fruit.

### 3.7. Total Flavonoid Content

Total flavonoid content was determined using a spectrophotometric method [51]. Briefly, 150 µL of extracts or catechin (Sigma-Aldrich, St. Louis, MO, USA) standards were mixed with 600 µL of distilled deionized water and 45 µL of 5% sodium nitrite solution. After 5 min, 45 µL of 10% aluminum chloride was added. The solution was mixed and incubated for 6 min at room temperature. Then, 300 µL of 1 M sodium hydroxide and 300 µL of distilled water were added and mixed. The absorbance was measured at 512 nm against the appropriate solvent blanks. The total flavonoid content was calculated based on the equation of the catechin calibration curve. The results are expressed as the arithmetic mean from three independent experiments in milligrams of catechin equivalents per 1 g of goji fruits.

### 3.8. Total Carotenoid Content

For carotenoid extraction, 1 g of powdered fruits was suspended in 10 mL of ethyl lactate, and 100 mg of α-tocopherol (Sigma-Aldrich, St. Louis, MO, USA) was added as an antioxidant [52]. The samples were placed in a water bath at 45 °C for 60 min, centrifuged (4500 rpm, 15 min), and the supernatants were collected. The absorbance was measured at 450 nm and 503 nm. The concentration of carotenoids was calculated according to the following equation [43];

C = 4.624 × A_450_ − 3.091 × A_503_, (2)

The results are expressed as the arithmetic mean from three independent experiments in milligrams of carotenoids per 1 g of goji fruits.

### 3.9. Parametric Index

To compare the quality of the tested dried goji berries, a parametric index was calculated based on the microbial contamination level and bioactivity profile, including the content of polyphenols, flavonoids, carotenoids, and antioxidant activity. Because the highest possible values are desirable in terms of the content of bioactive substances and the antioxidant capacity of the fruits, a 3-point scale was used, where 2 points were given to the highest value, 1 point to first statistically significantly different value lower from the highest value, and 0 points to the next lower value statistically different from the previous one. For microbial contamination, the inverse assignment was used—i.e., the higher the number of microorganisms, the lower the value of the assigned points while maintaining the same rules as above.

The calculation of the parametric index (*PI*) was conducted according to the following equitation:

(3) $$PI=\frac{a_{1}+a_{2}+\cdots +a_{n}}{n\cdot a_{max}}.$$

where *n* is the number of examined properties (*n* = 5), and *a*_1_, *a*_2_ *… a_n_* are the scores from the evaluation of each parameter (*a_max_* = 2).

### 3.10. Statistical Analysis

The results are expressed as the arithmetic mean ± standard deviation from three independent experiments. The significance of differences between means was determined using analysis of variance (one-way ANOVA) and Tukey HSD test, with *p* < 0.05 (Origin 8.1 software; OriginLab Corporation, Northampton, MA, USA).

## 4. Conclusions

This study compared the microbial diversity and content of bioactive compounds in dried goji berries available on the Polish market to those of the most highly valued goji berries from the Ningxia region in China. Differences were found, especially in terms of the quantitative and qualitative microbial structures of the berries. The microbial contamination was higher in goji berries available in Poland than from China, while the microbial diversity was similar in all samples. Given that the content of bioactive substances is a quality indicator of pharmacological relevance [12], the natural dried fruits from Ningxia region were of the highest quality (namely, a high content of polyphenols and high antioxidant activity), justifying their “daodi” status. Fruits grown in Poland showed the lowest antioxidant capacity and microbiological quality, although they contained significantly more carotenoids. The microbial diversity and biological activity of goji berries varied depending on both the country of cultivation and the method of drying, although it seems that the quality and quantity of the microbial populations depended mainly on the region of cultivation. Therefore, when evaluating the pro-health benefits and food safety, information about the origin of goji fruits may be of key importance for the consumer.

## Figures and Tables

**Table 1:** Antioxidant capacities and bioactive compounds content in goji berry extracts: G1—natural dried goji berries from China, packed in Poland; G2—freeze-dried goji berries from Poland; G3—natural dried goji berries from Ningxia, China; G4—freeze-dried goji berries from Ningxia, China.

Goji Berry Extracts		Antioxidant Capacities (μmol TE/g)	Total Phenolic Content (mg GAE/g)	Total Flavonoid Content (mg CAE/g)	Total Carotenoid Content (mg/g)
G1	water extract	M: 41.6 ^b, c^ SD: 0.2	M: 5.8 ^a^ SD: 0.6	M: 0.8 ^a, b^ SD: 0.1	M: 7.3 ^b^ SD: 0.1
	ethanol extract	M: 46.3 ^a^ SD: 1.1	M: 4.1 ^b^ SD: 0.6	M: 0.9 ^a^ SD: 0.2	
G2	water extract	M: 39.7 ^c^ SD: 0.2	M: 5.3 ^a, b^ SD: 0.1	M: 0.5 ^b^ SD: 0.1	M: 9.7 ^a^ SD: 0.3
	ethanol extract	M: 43.5 ^b^ SD: 0.5	M: 4.0 ^b^ SD: 0.3	M: 0.8 ^a, b^ SD: 0.1	
G3	water extract	M: 39.8 ^c^ SD: 0.9	M: 6.3 ^a^ SD: 0.3	M: 0.5 ^b^ SD: 0.1	M: 9.2 ^a^ SD: 0.7
	ethanol extract	M: 46.5 ^a^ SD: 1.6	M: 4.2 ^b^ SD: 0.9	M: 0.9 ^a^ SD: 0.2	
G4	water extract	M: 41.3 ^b, c^ SD: 0.4	M: 5.3 ^a, b^ SD: 0.4	M: 0.6 ^a, b^ SD: 0.1	M: 9.2 ^a^ SD: 0.4
	ethanol extract	M: 46.0 ^a^ SD: 0.4	M: 3.4 ^c, b^ SD: 0.2	M: 0.6 ^a, b^ SD: 0.1	

TE—Trolox equivalent; GAE—gallic acid equivalent; CAE—catechin equivalent; M—mean; SD—standard deviation. Means in a column followed by a common letter are not significantly different (Tukey’s test, *p* < 0.05).

## Data Availability

All data generated or analyzed during this study are included in this published article, any questions may be addressed to the corresponding author.
